# Editorial: Pituitary neuroendocrine tumors: tumorigenesis, pathogenesis, diagnosis and targeted therapy, from bench to bedside

**DOI:** 10.3389/fnins.2024.1358700

**Published:** 2024-02-29

**Authors:** Hao Lyu, De Ling Li, Yi Rong Yang, Yong Yao, Hui Wang, Guo Dong Huang

**Affiliations:** ^1^Shenzhen Key Laboratory of Neurosurgery, Department of Neurosurgery, The Shenzhen Second People's Hospital, First Affiliated Hospital of Shenzhen University, Shenzhen, China; ^2^Department of Neurosurgery, Beijing Tiantan Hospital, Beijing Neurosurgical Institute, Capital Medical University, Beijing, China; ^3^Department of Pharmaceutical Sciences, University of New Mexico Health Sciences Center, Albuquerque, NM, United States; ^4^Department of Neurosurgery, Peking Union Medical College Hospital (PUMCH), Chinese Academy of Medical Sciences and Peking Union Medical College (CAMS & PUMC), Beijing, China; ^5^Department of Neurosurgery, Third Affiliated Hospital of Sun Yat-sen University, Guangzhou, Guangdong, China

**Keywords:** pituitary, neuroendocrine tumor, tumorigenesis, pathogenesis, therapeutic targets

Pituitary neuroendocrine tumors (PitNETs) were previously classified as adenomas and accordingly referred to as pituitary adenomas. PitNETs have a lifetime prevalence of 5–10% in the general population. An adenoma, by definition, is a benign tumor that causes less harm than other types of tumors. In 2016, the International Pituitary Pathology Club renamed these tumors as neuroendocrine tumors (NETs) based on the hormone secreted by adenohypophysial cells. The classification of these tumors is based on their cell type, e.g., corticotroph, lactotroph, mammosomatotroph, thyrotroph, somatotroph and gonadotroph tumors. Some NETs, even small ones, may overproduce several kinds of hormones. Excessive hormone secretion may cause Cushing's disease, acromegaly, galactorrhea, hyperprolactinemia, reproductive problems and prolactinoma (Asa et al., 2021). However, some tumors are classified as silent corticotrophs or silent somatotrophs, or even as null cell tumors if they do not show a phenotype of pituitary lineage differentiation.

There have been five original research articles around this topic of interest, which focus on three major themes related to PitNETs:

1. Innovative visions for PitNET cell therapy;
2. Potential immunohistochemical biomarkers for the diagnosis and prognosis of PitNETs;
3. Cases of rare pituitary carcinoma and sellar xanthogranuloma.

The pathogenesis of most PitNETs is still poorly understood. Less than 5% of PitNETs have confirmed pathogenetic mutations. Li et al. introduced an animal model that used ovariectomy to remove endogenous estrogen, followed by implantation of a mini osmotic pump that released estradiol. They then identified involvement of the peroxisome proliferator-activated receptor gamma (PPARγ) pathway in the pathogenesis of lactotroph PitNETs. The study results showed reductions in the size and weight of lactotroph PitNETs after rats were given intranasal 15d-PGJ2, a PPARγ agonist. These results suggested a possible pathogenesis and a potential therapeutic target for lactotroph PitNETs.

PitNETs used to be incidental findings during autopsy or radiologic examination. With the increasing frequency of radiologic examinations, PitNETs are increasingly being diagnosed. An estimated 20% of pituitary studies identify small tumors, most of which are nonfunctioning. Therefore, sensitive and specific biomarkers are needed for the diagnosis of PitNETs. Zhang et al. compared OCT3/4 with PLAP as biomarkers for the diagnosis of intracranial germ cell tumors (iGCTs). They elucidated the importance of OCT3/4 expression in the prognosis of individuals with iGCT, and proved that OCT3/4 and PLAP are promising immunohistochemical markers for the diagnosis and prognostication of iGCTs.

Lactotrophs are the most commonly identified PitNETs. They cause hyperprolactinemia, galactorrhea, infertility, amenorrhea, headache and visual impairment. The mechanism of lactotroph tumorigenesis at the molecular level is still unclear. A large body of research has revealed that autophagic cell death is deeply involved in PitNET treatment. Li et al. proved that intranasal administration of the PPARγ agonist 15d-PGJ2 can induce apoptotic and autophagic cell death, resulting in the inhibition of rat lactotroph growth through reactive oxygen species-dependent activation of transcription factor EB and downstream autophagy regulation.

The Research Topic focuses on several aspects of PitNETs, from molecular mechanisms to clinical cases, and on descriptions of new potential therapeutic targets. PitNET research comprises a wide range of topics and involves many different scientific fields. This Research Topic aims to help researchers and clinicians in understanding aspects of PitNETs, including potential therapeutic targets, mechanisms of tumorigenesis, cases in real life and how they are treated in the clinic, and to provide more possibilities for future PitNET studies.

## Author contributions

HL: Writing – original draft. DL: Writing – review & editing. YRY: Writing – review & editing. YY: Writing – review & editing. HW: Writing – review & editing. GH: Writing – original draft, Writing – review & editing.
